# Improving the Retrieval of Arabic Web Search Results Using Enhanced *k*-Means Clustering Algorithm

**DOI:** 10.3390/e23040449

**Published:** 2021-04-11

**Authors:** Amjad F. Alsuhaim, Aqil M. Azmi, Muhammad Hussain

**Affiliations:** 1 Department of Computer Science, College of Computer & Information Sciences, King Saud University, Riyadh 11543, Saudi Arabia; amjadf.cs@gmail.com (A.F.A.); mhussain@ksu.edu.sa (M.H.); 2 National Center for Telecommunication and Defense Systems Technologies, King Abdulaziz City for Science and Technology, Riyadh 12354, Saudi Arabia

**Keywords:** Arabic, clustering algorithms, web search, enhanced *k*-means, information retrieval, text mining

## Abstract

Traditional information retrieval systems return a ranked list of results to a user’s query. This list is often long, and the user cannot explore all the results retrieved. It is also ineffective for a highly ambiguous language such as Arabic. The modern writing style of Arabic excludes the diacritical marking, without which Arabic words become ambiguous. For a search query, the user has to skim over the document to infer if the word has the same meaning they are after, which is a time-consuming task. It is hoped that clustering the retrieved documents will collate documents into clear and meaningful groups. In this paper, we use an enhanced *k*-means clustering algorithm, which yields a faster clustering time than the regular *k*-means. The algorithm uses the distance calculated from previous iterations to minimize the number of distance calculations. We propose a system to cluster Arabic search results using the enhanced *k*-means algorithm, labeling each cluster with the most frequent word in the cluster. This system will help Arabic web users identify each cluster’s topic and go directly to the required cluster. Experimentally, the enhanced *k*-means algorithm reduced the execution time by 60% for the stemmed dataset and 47% for the non-stemmed dataset when compared to the regular *k*-means, while slightly improving the purity.

## 1. Introduction

No doubt, the World Wide Web is the largest source of information. According to an IBM Marketing Cloud study, 90% of the data on the Internet has been created since 2016, about 3 quintillion (3 billion GB) bytes of data per day [1], and by one estimate, it is predicted to grow to 463 quintillion bytes per day in 2025. This ever-growing data poses a challenge, even for a simple task as information retrieval (IR). This is where the search engines come to the rescue. Google, one of the most popular and widely used search engines, is queried several billion times every day. During early June 2020, while writing this section, there were 83,379 Google searches every second, or 300M searches per hour. According to a 2012 McKinsey report, employees spend 1.8 h every day searching and gathering information, which translates to 9.3 h per week on average [2]. Nicely put in the report, “businesses hire 5 employees but only 4 show up to work; the fifth is off searching for answers, but not contributing any value”. In another survey, only one in five users is lucky in their first search, while for the rest, they may take up to eight searches to find the right document and information [3]. This paper aims to tackle the web search for a better, more efficient, and faster experience.

The search engines allow users to specify queries as a simple list of keywords. However, this list of keywords is not a good descriptor of the needed information. In response to a user’s query, search engines display pages of results, popularly known as Search Engine Result Pages. Though results are usually ranked by relevance to the query [4], it still is ineffective, especially if the query is ambiguous. Typically, users browse the top results on the first page; hence, it is very likely they may miss some of the relevant documents. The problem of improving search engine results and obtaining the desired information has been processed in different ways. Clustering the search engine results is one such way [5].

Clustering algorithms group a set of documents into subsets or clusters. It is an unsupervised process with no human intervention. The goal is to create clusters that are coherent internally but clearly distinguishable from each other. The documents within a cluster should be as similar as possible, and documents in one cluster should be as dissimilar as possible from documents in other clusters [5]. The first step in clustering results is to process the language naturally so that the machine can better understand the linguistic structure automatically [6].

Clustering helps users search a large set of documents more efficiently by reducing the search domain and time. For instance, in traditional retrieval systems, the search results for the term, say “Mac” can return various documents related to Apple OS, a beauty product, the fast-food restaurant McDonalds, among others. Clustering the search results will help the user go directly to the required cluster. Clustering has been around for a while, and many studies have contributed different techniques to cluster documents. Most of the previous works focused primarily on clustering English documents; other languages were less fortunate, for example, Arabic.

With a population of around 445 million, Arabic language users constitute the fastest-growing language group on the web with regard to the number of Internet users. In www.internetworldstats.com/stats7.htm accessed on 6 April 2021, during the twenty-year period ending March 2020, Arabic language Internet users grew by 9348%. Russian language users were a distant second with a growth of 3653.4% in the same period. Since the Internet penetration for the Arabic users is 53%—one of the lowest—signaling this percentage is likely to grow further. The Internet penetration is the ratio between the sum of Internet users speaking a language and the total population estimate that speaks that specific language.

One of the main problems in Modern Standard Arabic (MSA) is the lack of diacritical markings in the written text. These markings disambiguate the meaning and the sense of a word. Their lack is a major source of the ambiguity of Arabic text. For example, the word (ذهب: *hb) could either be “gold”, or “went”. For convenience, we will be using Buckwalter transliteration scheme for those having difficulty recognizing the Arabic script. The entire scheme is at www.qamus.org/transliteration.htm accessed on 6 April 2021. These two are the two more popular meanings, but there are others as well. Had it been vowelized, say (ذَهَبْ: *ahabo), then we will immediately read it “gold”. We will leave further details to Section 2.

Given the importance of clustering in information retrieval (IR), we would like to study the impact of a new clustering algorithm on Arabic IR. Some works looked at some clustering techniques (e.g., *k*-means algorithm) to cluster Arabic search results. However, there is always room for enhancement. In general, the research in Arabic NLP lags behind many other languages and certainly does not match Arabic language users’ explosive growth in internet usage. Summarizing our contributions:

- We compile a suitable dataset to test clustering algorithms. Each word in the dataset has multiple meanings; thus, it is hoped that clustering will collate all documents having a word with the same meaning.
- We investigate the enhanced *k*-means algorithm on Arabic IR, including the impact of the stemming process.
- Compare the performance of enhanced *k*-means, the regular *k*-means algorithms on the dataset, and how their performance measures are impacted by the stemming process. Results significance were confirmed using paired *t*-test.
- Surveyed users for their preference in doing a web search.

The rest of this paper is organized as follows: Section 2 provides background about Arabic language properties and a brief look at clustering. A look at related works on clustering Arabic and non-Arabic texts is in Section 3. In Section 4, we describe our proposed system. We look at the experiments and discuss the results in Section 5, and we finally conclude in Section 6.

## 2. Background

In this section, we briefly delve into the challenges due to the nature of the Arabic language, clustering and the metrics used to assess it.

### 2.1. Challenges in Arabic IR

Arabic poses many challenges for information retrieval; most of the challenges are due to orthography and morphology [7]. The Arabic orthographic system uses small diacritical markings to represent different short vowels. There are a total of thirteen different diacritics, and these are placed either above or below the letter to indicate the phonetic information associated with each letter to clarify the sense and meaning of the word [8]. Below we go over some of the characteristics of the Arabic language that may cause potential problems with Arabic IR.

**Figure 1:**
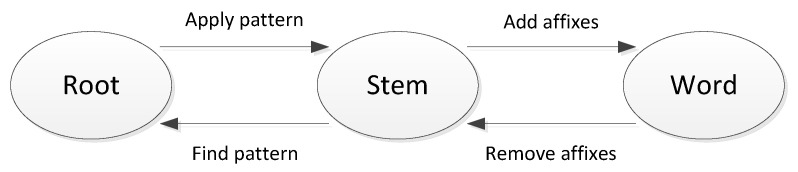
General Arabic word construction system. In Arabic, the affix could be any combination of prefix(es) and suffix(es).

- Ambiguity. In Arabic, words with similar spelling may have different pronunciations and meanings that can only be determined by the context and proper knowledge of the grammar. However, when ambiguity persists, it is resolved through the diacritical markings. Unfortunately, in the modern writing system the diacritics are not written, as it is assumed—erroneously—the reader will disambiguate the meaning. Azmi and Almajed [8] have shown this is far from the truth and that ambiguity is a serious problem in MSA as the problem of finding the proper semantic meaning of a given word is a non-trivial task. Just to give an idea, a single undiacritized word (عقد: *Eqd*), could be any of the following, “necklace”, “knots”, “contract”, “decade”, “pact”, and “complicated”. A study showed that for each undiacritized word, it is possible to have (on average) 11.6 different interpretations/meanings [9]. Farghaly and Shaalan [10] reported on a firm that has been working on machine translation for the last 50 years; they saw as many as 19.2 ambiguities for a token in MSA, while for most languages, it was on average 2.3.
- Arabic morphology, complex yet systematic. The nouns and verbs are derived from roots by applying templates, thereby generating stems. Applying templates often involves introducing infixes or deleting or replacing letters from the root. We may also join multiple prefixes and/or suffixes to a stem to form a word. Prefixes include prepositions, determiners, and coordinating conjunctions, while suffixes include attached pronouns, gender indicator, and number markers [7]. The most common Arabic root has three consonants (triliteral roots), and the largest one has five-consonants (quinquiliteral roots). The consonant root can be viewed as a core around which are clustered a wide array of potential meanings, depending on which pattern is keyed into the root [11]. The number of lexical roots in Arabic has been estimated to range between 5000 and 6500 [11].
  Figure 1 shows the general word construction system in Arabic. Table 1 provides an example of a complex Arabic word with different affixes, a simple example of how morphology may impact retrieval. One of the very common prefixes in Arabic is the definite article (ال: *Al*) “the”, always prefixed to another word and never stands alone. This leads to clustering a large number of alphabetically grouped documents in the index file.
- Irregular (or broken) plurals, for example, leaf → leaves (in English). In Arabic it is more common. About 41% of the Arabic plurals are broken, constituting about 10% of the text in large Arabic corpora [12].
- Out of vocabulary (OOV) words, such as a named entity or technical term. The OOV is a common source of error in any retrieval system. In [13], it was reported that half of the OOV words in Arabic are a named entity. One study reported 15 different spellings for Condoleezza (former US Secretary of State), with four different ones found on the CNN-Arabic website alone [12].

### 2.2. Clustering

Clustering solves a problem that may arise when a term has different meanings, such as “Jaguar” or “MAC”. The word “Jaguar” could be a car brand or an animal. It could also refer to a specific version of the Mac Operating System, in particular Mac OS X 10.2, which was marketed as Jaguar. Clustering (or cluster analysis, taxonomy analysis) is an unsupervised process that divides a set of objects into homogeneous groups based on some similarity measure. The difference between clustering and classification is that in the former the classes are also to be defined, while in the latter they are predefined.

**Figure 2:**
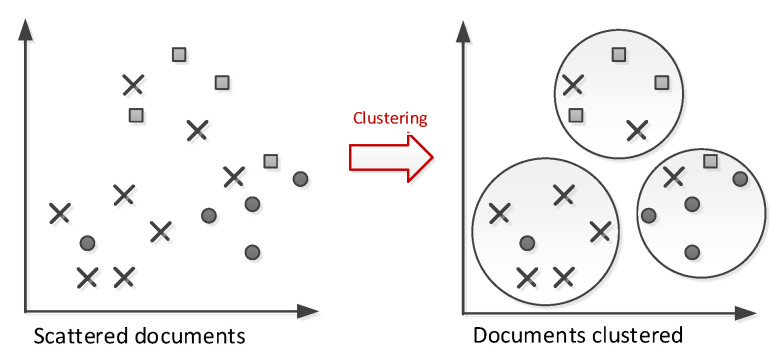
Example of a possible clustering into 3 clusters.

The typical goal in clustering is to attain high intra-cluster similarity and low inter-cluster similarity. That is, we want documents within a cluster to be as similar as possible and documents from different clusters to be dissimilar. Figure 2 shows scattered documents and one possible clustering. Therefore, it is hoped that all documents that refer to Jaguar the animal are in a single cluster, and those that refer to a car brand in another cluster.

The *k*-means is one of the most used clustering algorithms, first described by [14,15]. In this algorithm, the number of clusters *k* is assumed to be fixed. The basic idea is [16]: given an initial (not optimal) clustering, move each point to its new nearest center, update the clustering centers by calculating the mean of the member points, and repeat the relocating-and-updating process until we reach the convergence criteria (e.g., predefined number of iterations, or minimize the objective function).

Suppose we have a set of *n* data points (documents) $X=\{x_{1},x_{2},\ldots ,x_{n}\}$, and let $\Omega =\{\omega_{1},\omega_{2},\ldots ,\omega_{k}\}$ be the *k* clusters, where k≤n. One possible objective function is the sum of the squared distances to the cluster centers. Formally, we can express this as Equation (1):

(1) $$\mathrm{minimize}\sum_{j=1}^{k}\sum_{i=1}^{n}{∥x_{i}^{\left(j\right)}-\omega_{j}∥}^{2},$$

where $x_{i}^{\left(j\right)}$ is a data point that belongs to cluster $\omega_{j}$. The computational time complexity of the *k*-means algorithm is O(nkI), where *I* is the number of iterations till stopping criteria are reached.

Some of the measures to assess the quality of clustering are purity and entropy. Another is accuracy, which is typically used for evaluating the performance of classification. Purity is an external evaluation criterion for measuring the quality of the cluster. It measures the extent to which a cluster contains objects of a single class [5]. To compute *purity*, each cluster is assigned to the class in which it is the most common. Then the accuracy of this assignment is measured by counting the number of correctly assigned data points and divided by the total number of data points (*n*). Formally, we express *purity* as follows,

(2) $$Purity\left(\Omega ,C\right)=\frac{1}{n}\sum_{i}\max_{j}\left|\omega_{i}\cap c_{j}\right|,$$

where Ω is the set of clusters, and $C=\{c_{1},c_{2},\ldots \}$ is the set of classes. A perfect clustering will have a *purity* of one, while it will be close to zero for a bad clustering. In Figure 2 we have three objects {×,∘,□}. In cluster 1, the majority object is × occurring five times, and ∘ for cluster 2 and it occurs four times, etc. The *purity* for the clustering in Figure 2 is (5+4+3)/17=0.706.

Entropy measures the degree to which each cluster consists of objects of a single class. The entropy of cluster ω is defined as ([17] pp. 487–568),

(3) $$Entropy\left(\omega \right)=-\sum_{c\in C}\mathrm{Pr}\left(\omega_{c}\right)\log_{2}\mathrm{Pr}\left(\omega_{c}\right),$$

where $\mathrm{Pr}(\omega_{c})$ is the probability of an object being classified as *c* in cluster ω, which simply equals the count of objects classified as *c* in cluster ω over the number of objects in cluster ω. The total entropy of a clustering is calculated as the sum of the entropies of each cluster weighted by the size of each cluster,

(4) $$Entropy\left(\Omega \right)=\frac{1}{n}\sum_{\omega \in \Omega }\left|\omega \right|\cdot Entropy\left(\omega \right).$$

For Figure 2, the entropy for cluster $\omega_{1}$ is 1.179, while $Entropy(\omega_{2})=0.821$. The total entropy is 0.45. An entropy of zero means a perfect clustering.

One of the drawbacks of *k*-means clustering is the need to predefine the value of *k*, the number of clusters. Finding the optimal number of clusters is not a trivial task nor has a clear answer. It is kind of subjective and relies on the method used in measuring similarities. The method of finding the optimal value of *k* can be divided into direct methods or statistical testing methods. The direct method is based on criterion optimization, such as within-cluster sum of squares (elbow method), and the average silhouette method. The statistical testing methods are based on comparing evidence against the null hypothesis, such as gap statistics.

When defining clusters, our goal was to minimize the total intra-cluster variations (or total Within-cluster Sum of Square (WSS)). The WSS measures the compactness of the clustering, and we want to minimize it. The Elbow method looks at the total WSS. It picks the smallest WSS value such that adding another cluster has little effect on the total value of WSS. The optimal number of clusters corresponds to the smallest WSS.

The average silhouette method calculates the average silhouette, which measures how well each object lies in its cluster for different *k* values. A higher value means a good clustering result. The optimal number of clusters is the one that maximizes the average silhouette over a range of possible values for *k* ([18] pp. 68–125).

The gap statistic compares the total WSS for different values of *k* with their expected values under the null reference distribution of the data. The estimate of the optimal clusters will be a value that maximizes the gap statistic.

The gap statistic was devised by [19]. It calculates the WSS of all objects from the cluster center. This is known as dispersion. The algorithm creates a sample dataset of the original and calculates its mean dispersion of the sample dataset. Every gap is described as a logarithmic difference between the mean dispersion of reference datasets and dispersion of the original dataset [20]. The gap is maximized when applying the minimum value of *k*.

## 3. Related Work

In this section, two aspects of previous works related to clustering are highlighted: the Arabic and non-Arabic domains. We start by covering related works in the non-Arabic domain, as those in the Arabic domain are few.

### 3.1. Non-Arabic Text Clustering

The first post-retrieval system was proposed in [21,22,23], which is the Grouper clustering interface to the HuskySearch meta-search engine for the English language. The authors explained that the advantage of Grouper is that it is independent of the search engine. The benefits of an independent interface are that coherent clusters are obtained, the browsing is more efficient, and the clustering speed is faster. Grouper’s clustering algorithm is the Suffix-Tree Clustering (STC) algorithm, which groups retrieved documents based on common phrases. Overlapping between clusters is allowed in Grouper. In [22,23], the clustering search engine Vivisimo was described as the most popular commercial clustering search engine in 2000. It produced high-quality hierarchical taxonomies based on search results. The cluster labels in Vivisimo are composed of phrases, which use a specially developed heuristic algorithm based on artificial intelligence.

Fahim et al. [24], proposed an enhanced *k*-means algorithm. The standard *k*-means algorithm calculates the distance between the points and all cluster centers in each iteration, which is computationally expensive. They, therefore, developed a way to use distance information in the previous iteration to reduce computational time and cost. The authors tested their algorithm on different datasets and compared the performance with the standard *k*-means algorithm. The experimental results confirmed that the enhanced *k*-means surpassed the standard *k*-means algorithm in both execution time and cluster quality.

In [25], the authors combined hierarchical and *k*-means clustering algorithms to present a new system called CONDOR. It is a hierarchical document clustering system that uses the *k*-means algorithm. They compared their method with the regular method, in which the initial centroids had been predefined. The results showed that the performance improved greatly. The limitation was that the system did not achieve optimal clustering, and it was slow. Cheng et al. [26] improved the *k*-means algorithm’s performance by using the local search mechanism to obtain the local extreme point, which optimized the objective function. It then jumped to that point and increased the quality of the solution. The results indicated that this method provides better performance than the standard algorithm without affecting the clustering speed. The quality of clustering showed clear improvement in small datasets but limited improvement in large datasets. These results might have relied on the neighborhood, which was not sensitive to the disturbance of the objective function.

Bide and Shedge [27] presented an improved document clustering algorithm that did not require a predefined value for *k*. Instead, it used cluster labels as the input and a cosine similarity measure to group similar documents into a suitable number of clusters. The experimental results indicated that the accuracy of the proposed algorithm was high compared with the existing similar algorithm based on the *F*-measure. Gupta et al. [28] used *k*-means clustering to study an outlier problem. They tried to minimize the variance between data points in the same cluster while ignoring a small set of a point that could be labeled as an outlier. The authors proposed an algorithm for *k*-means clustering with an outlier. The algorithm is simple, practical, and can be adapted to scale large data. They evaluated the performance in synthetic and large-scale real-world data, including SUSY, Power, Skin, Shuttle, and Covertype; these datasets had 5M, 2M, 245K, 43K, and 11K instances, respectively. The experimental results showed that the proposed algorithm was accurate. In the synthetic dataset, recall and precision were one, while in the real dataset, one was obtained in recall and precision by most.

The following works are not related to IR but show interesting applications of clustering. Typically, data clustering algorithms are effective in mining information from large data that is offline; in other words, they are not suitable for clustering online data streams. Chen et al. [29] proposed FGCS, a novel grid-based clustering algorithm for a hybrid data stream. While [30] clustered users of social media based on their time-varying topic distributions. They proposed a swarm optimized cluster-based framework to retrieve user-specific knowledge from a collection of documents. Initially, they grouped documents using a bio-inspired K-Flock clustering algorithm, which is followed by extracting frequent patterns from each cluster. Finally, the authors use a probabilistic model based on cosine similarity to retrieve query-specific documents from clusters.

### 3.2. Arabic Text Clustering

Froud et al. [31] examined the impact of stemming on clustering Arabic documents. The study used five similarity distance measures and two stemmers (i.e., Khoja’s stemmer and Larkey’s stemmer). The results showed that Euclidean distance, cosine similarity, and the Jaccard measures provided more efficient results without stemming, which led to more coherent clusters. The Pearson correlation and averaged Kullback–Leibler divergence yielded slightly better results in stemming than the three measurements did. The results showed that using stemming with the documents resulted in less document representation and a faster clustering process. The dataset used was the Corpus of Contemporary Arabic (CCA) [32], which has 12 categories.

In [33], the stemming effect on clustering Arabic documents was examined using the *k*-means algorithm. The training dataset contained 1445 Arabic documents in nine distinction categories. The experimental results showed that stemming decreased accuracy because it led to incorrectly distinguished documents. In the best stemmed result, 55% of the documents were successfully categorized; and it goes up to 69% for the non-stemmed results. Ghanem and Ashour [34] examined some stemming techniques in clustering the Arabic language to determine whether they improved performance. The three techniques used were root-based stemming, light stemming, and non-stemming. The *k*-means algorithm was used to cluster the documents and evaluate the effects on recall, precision, and F-measure. The experiment results showed that light stemming outperformed the other stemming in all three measures. The dataset used was Open Source Arabic Corpora (OSAC) [35].

Sahmoudi and Lachkar [36] found that it was impossible to apply the STC algorithm directly to the Arabic language because of its properties. Therefore, they integrated STC into a new scheme. They proposed an Arabic web search results clustering (WRC) system, which clustered Arabic search results retrieved from Google application programming interface (API) using the integrated STC algorithm. The experimental results indicated that the proposed approach was effective and efficient, facilitating Arabic users to quickly browse through search results. The authors compared their results with three existing WRC systems: IBoogie, Clusty, and Yippy. Clusty is the successor to Vivisimo, which was later sold to Yippy, Inc. These web post-retrieval systems were developed for the Latin or cross-languages that generate different clusters using different clustering algorithms. Later, this work was further enhanced in [37]. The interactive system enabled the user to quickly decide whether a cluster’s content is interesting without reformulating the query by just choosing the most accurate label for the information in the hierarchy. The experimental results showed that the quality of the cluster labels was high, and they could help the user reformulate the query. Moreover, the generated label hierarchy gave the user a sense of generalization or specialization. In [38], the same authors further improved their system by applying a formal concept analysis (FCA). An Arabic WRC system was integrated into web documents based on FCA, which used conceptual clustering, a new method for clustering web search results and solving the web browsing problem, specifically for European languages. They evaluated their system using Google and Bing search APIs and reported encouraging and efficient results.

Alghamdi et al. [39] proposed an improved *k*-means algorithm to first extract semantic similarity using Arabic VerbNet lexicon [40]. Then, results were clustered based on the semantic similarity, and the annotations were added. The experimental results showed that the purity of the cluster has increased, while the mean intra-cluster distance has decreased. The corpus examined in the experiment was a collection of newspapers, including Al-Akhbar news (http://www.al-akhbar.com/ accessed on 6 April 2021), Alhayat news (http://alhayat.com/ accessed on 6 April 2021), Aldostor news (http://dostor.org/ accessed on 6 April 2021), Alriyadh news (http://www.alriyadh.com accessed on 6 April 2021), and others. The corpus contained 753 documents in six categories. They used Easy Web Extract to extract the data gathered from these newspapers.

In [41], a hybrid clustering method (*k*-means and hierarchical) was proposed for Arabic text summarization of single and multi-documents. The model grouped the documents into several clusters and then extracted the key phrase from each cluster. The key phrase assisted in identifying important sentences and also locating similar sentences. From each group of similar sentences, the model picks one sentence that is above a predefined threshold. The other sentences in the group are ignored. For a similarity measure, they used cosine similarity and the Jaccard coefficient. The experimental results showed increased precision, recall, and F-measure in single and multi-documents. The corpus used was the Essex Arabic Summaries Corpus (EASC). Available for free download from https://sourceforge.net/projects/easc-corpus/ accessed on 6 April 2021. The main limitation was that they did not compare their work with other studies.

Abuaiadah [42] studied the bisect *k*-means clustering and compared its performance with the standard *k*-means algorithm for clustering Arabic documents. They used three stemmers and five similarity and distance functions: Pearson correlation coefficient, cosine, Jaccard coefficient, Euclidean distance function, and average Kullback–Leibler divergence. The experimental results showed that the bisecting *k*-means algorithm achieved better purity than the standard *k*-means. The removal of stop-words clearly improved the bisecting *k*-means algorithm results, but only a small improvement in the case of the *k*-means algorithm. The dataset used in the experiment is from [43]. It had nine categories, each of which contained 300 documents. In [44], the authors grouped Arabic clustering into three categories. The first challenge was the difficulty in finding a significant term to represent the original content. The second challenge was in reducing the data dimensionality without losing the meaning of the data. The third challenge was designing a clustering model to improve clustering performance. Moreover, they presented a brief explanation of the existing Arabic web page clustering method to clarify existing problems and examine the features selected to overcome clustering difficulties.

In [45], three approaches were proposed: unsupervised, semi-supervised, and semi-supervised with dimension reduction to construct a clustering based textual classification of Arabic documents. For clustering they used *k*-means, incremental *k*-means, *k*-means with threshold, and *k*-means with dimension reduction. The dataset consisted of five categories collected from two different online news sources. The results were evaluated using F-measure, entropy, and accuracy. The experimental results showed their proposed system yielded better accuracy when compared to other systems. Moreover, increasing the ratio of reduction can ruin important terms.

## 4. Proposed System

In this section, we introduce the proposed system for clustering the results of Arabic web searches. Our objective is to cluster similar documents, hoping that it will improve and enhance the quality of the retrieved results.

**Figure 3:**
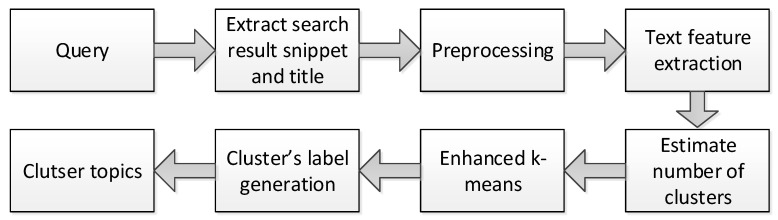
The architecture of our proposed system.

Figure 3 shows the architecture of our proposed system. There are seven stages: extracting a snippet from the search engine, preprocessing the snippet text, extracting text features, estimating the number of clusters, applying the enhanced *k*-means clustering algorithm, evaluating clusters, and creating clustering labels. In the extracting stage, we use two methods to extract snippets and titles from the search engine—in our case, Google. In the first method, Google APIs are used, which is limited to only 100 search results. For the other method, data extraction tools, such as Data Miner, are used, which can be customized to extract all Google search result pages. Both methods produce an Excel sheet containing the search result’s title, URL, and snippet. After the extraction, we preprocess the result’s title and snippet. The preprocessing phase includes two steps: (a) removing English letters, numbers, and repeated characters; and optionally (b) stemming the text using ISRI (Information Science Research Institute) Arabic stemmer [46]. The ISRI stemmer has many features in common with the Khoja stemmer [47], a root-based stemmer. However, unlike the Khoja stemmer, it does not need a root dictionary, which is the main difference.

Stemming is a computational procedure that reduces all words with the same root (or the same stem, in case prefixes are left untouched) to a common form, usually by stripping each word of its derivational and inflectional suffixes, for example, the words “retrieval”, “retrieved”, “retrieves” are reduced to the stem “retrieve”. In IR, grouping words with the same root (or stem) increases the success with which documents can be matched against a query [48].

Following the extraction and preprocessing of the snippet text, each document (title, URL, snippet, etc.) is stored in an Excel sheet row. The textual data cannot be fed to the clustering algorithm directly, so we need the *tf-idf* (short for term frequency-inverse document frequency) method to convert the textual data to numerical data. This method is used to represent documents in most data mining and information retrieval applications due to its properties [49]. It assigns a numerical weight to every term in a collection of documents so that the clustering algorithm can process it. Furthermore, it is useful in labeling each cluster with the most frequent terms in that cluster. Before applying the algorithm, we needed to determine the appropriate value of *k*, the number of clusters. This is necessary in most partitioning clustering algorithms, for example, *k*-means algorithm.

The process of finding the optimal value for *k* is not an easy task (see Section 2.2). It is subjective and relies on the method used in similarity measuring and parameter partitioning. Hence, we tried to optimize the number of clusters by running the gap statistic method to obtain an initial value for *k*. Then, we applied the elbow method to improve the accuracy of the results in the range of k−2 to k+2. We chose the number to which adding another cluster would have the smallest effect on the Within-cluster Sum of Square (WSS) value. The optimized number of cluster centers *k* is used in the main clustering algorithm (Algorithm 1). The enhanced *k*-means algorithm was used to cluster English language documents [24], and we adapted it for the Arabic language documents. The first step is to randomly generate *k* centroids and then calculate the distance from each point to all the centroids. Then, the point is assigned to the nearest centroid. Each centroid is updated to point to the center of its cluster, and the distance between the point and the new centroid is recalculated. If it is less than the old distance, it is assigned to that cluster. Otherwise, the distance between the point and the rest of the centroids is calculated, and it is assigned to the nearest one. The process is repeated till there is no change, i.e., the centroids did not move. At which the points and the cluster number is returned. The complexity of Algorithm 1 is O(nk), where n=|X| is the number of data points, and *k* is the number of clusters.
**Algorithm 1:** The enhanced *k*-means algorithm.
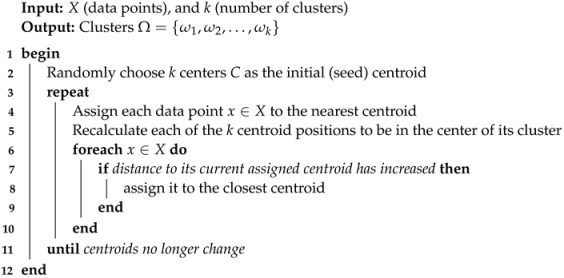


Each cluster is labeled with the five most frequent terms in the cluster, which are calculated using the *tf-idf* matrix. These labels indicate the meaning of that cluster.

## 5. Results and Discussion

In this section, we will describe the different experiments and discuss their results. Things covered: (a) how we compiled the dataset; (b) experiments to estimate the number of clusters; (c) performance comparison between *k*-means, and the enhanced *k*-means clustering algorithms with and without stemming; (d) generating labels for the clusters; and finally (e) comparison between our proposed system and similar ones. All the experiments were executed on a laptop using Intel i7-2.5 GHz CPU and an 8 GB RAM. The system was implemented using python 3.7.

### 5.1. The Dataset

Our main objective by clustering the search results is to make it easier for the user to reach the desired result. As we have seen earlier, ambiguity is more common in Arabic than in other languages (see Section 2.1). Given this, we prepared the dataset as follows. We manually compiled a set of fifty Arabic terms, each having more than one meaning. Table 2 lists all these terms.

For example, one of the words in the list is (شعر: *$Er*). Among its meanings are “hair”, “poetry”, “felt” (v.), “poets”, etc. Using each term, we did a simple search query and picked the top returned results (as returned by the search engine). On average, 130 search results per term. Thus, our dataset is comprised of 6500 results. These were saved in an Excel sheet, one result (i.e., title, URL, and snippet) per row. We then sought human experts to classify the search results based on their meanings.

### 5.2. Estimating the Number of Clusters

The next step is to estimate the number of clusters of each term’s search result. The selection of the κ cluster, which represents how many meanings each word has, could be divided into different abstraction levels. However, to get the best division, we manually classified the words’ meaning by reading the extracted snippet. Then, we calculated how many meanings the word has in the search result as κ. Finally, we sought an Arabic specialist to review each word’s division.

Then, we started with gap statistics to get an initial value for the number of clusters, and followed with the elbow method to get an optimal number of clusters. We applied the gap statistics method and part of the python library, which returned a suitable value for the number of clusters, κ. Of the 50 terms examined, 19 terms were correctly answered (i.e., hard clustering). The accuracy results for this system were 38%. If, on the other hand, we consider answers having a small difference of one or two clusters as an acceptable answer, then 40 terms were correctly answered (soft clustering). The results for accuracy jumps to 82% (see Table 3). In hard clustering, each document belongs to a single cluster, whereas in the soft clustering it may belong to more than one cluster.

In the elbow method, adding the author cluster has a small effect on the WSS value, which is the suitable number of clusters. We applied the elbow method in the range of (κ−2,κ+2), where κ is the value returned by the gap statistics method. Following this, 30 terms were correctly answered (hard clustering), which increased the accuracy to 60%. For soft clustering, 47 terms were correctly answered, and the accuracy increased to 94%; see Table 3.

**Figure 4:**
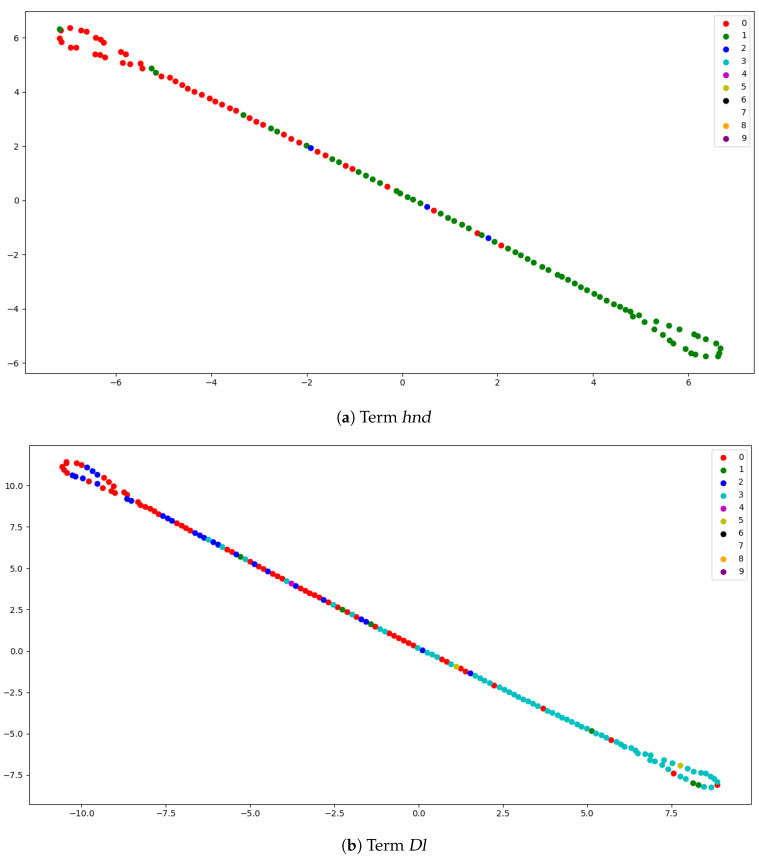
Visualization of two sample terms following t-Distributed Stochastic Neighbor Embedding (t-SNE) reduction.

The t-Distributed Stochastic Neighbor Embedding (t-SNE) is used to reduce the data dimension into two dimensions to visualize it. The t-SNE produces a reduced feature space in which similar points are modeled by nearby points and dissimilar points are modeled by distant points with high probability. We used t-SNE to reduce the *tf-idf* matrix dimension and then plotted it. Figure 4 presents a t-SNE visualization of two sample terms from Table 2. Each point represents a document and each color represents a cluster.

### 5.3. Labeling Clusters

After clustering the search results, we labeled the clusters. It is a crucial step because it kind of gives meaning to the data in the cluster. For instance, the term (ذهب: *hb) returned 133 search results. The results were classified into three groups based on meaning (in the absence of diacritics). Table 4 shows the cluster labels that were generated automatically by the proposed system. The first cluster (cluster no. 0) had the meaning of “gold”, and the second (cluster no. 1) had the meaning of “went”. The meaning of the third cluster was undefined because it included outliers unrelated to the term. The term score is the term’s weight in the *tf-idf* metric grouped for each cluster.

### 5.4. Performance of Enhanced k-Means

We will report the performance of enhanced *k*-means algorithms on the dataset, with and without using the stemming. For comparison, we will use three measures: purity, entropy, and the execution time.

**Figure 5:**
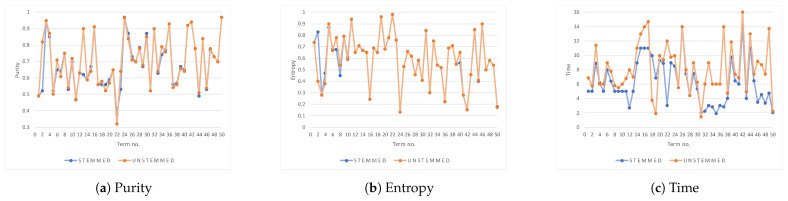
The performance of the enhanced *k*-means algorithm with and without the stemming.

Table 5 lists the performance results on selected Arabic terms from the dataset using the enhanced *k*-means algorithm, with and without the stemming. For all a full picture, Figure 5 plots the purity, entropy, and the time (in seconds) for the enhanced *k*-means algorithm with and without the stemming for each of the fifty terms in the dataset (Table 2).

Visually, we can tell that the stemming had a negligible impact on the purity and the entropy of the clustering; however, it did speed up the clustering process. We would like to confirm this result statistically using the paired *t*-test. To compare two paired values where both observations are taken from the same subjects, we can perform a paired *t*-test. We will be pairing the difference in performance (e.g., purity) of clustering each term with and without stemming for all 50 terms. Our null hypothesis is: there is no difference; in other words, stemming does not impact the individual performance measure.

Table 6 summarizes the paired *t*-test for the difference in three performance measures for clustering. We reject the null hypothesis if the absolute value of *t* is at least, if not more extreme than $t_{\mathrm{crit}}$, i.e., $\left|t|\geq |t_{\mathrm{crit}}\right|$. First, a paired *t*-test was performed to determine if stemming is effective on the purity using the enhanced *k*-means algorithm. The mean of the paired differences in purity is (mean = 0.0118, standard deviation = 0.0626, N = 50) was statistically insignificant, as $t=1.3314<t_{\mathrm{crit}}=2.0096$. Similarly, looking at entropy, we conclude that stemming had an insignificant (statistically) impact on the entropy. However, stemming is effective (statistically significant) in improving the clustering time. With no stemming, the mean time to cluster is 8.2088 s (average for 50 terms), and it drops to 6.229 s when stemming is used. This means, by stemming, we improved the speed of clustering on average by 33%, all at the same time with no significant change in purity and entropy.

Furthermore, we calculated the average distance of all the data points in a cluster from its centroid and the distance between all the centroids for both cases (i.e., with and without stemming). We designated these two distances $d_{1}$ and $d_{2}$, respectively. Table 7 shows the distance for selected Arabic terms. Again, we would like to confirm if stemming had an impact on the distance using the paired *t*-test. Let our null hypothesis be: there is no difference. Calculating using α=0.05, the 1-tailed *p*-value for $d_{1}$ is 0.047, and is 0.045 for $d_{2}$. Both results are statistically significant. This means that stemming did reduce (with statistical significance) the distances. However, from the practical point of view, we would prefer to reduce the $d_{1}$ distance and retain (if not increase) the $d_{2}$ distance.

### 5.5. Comparison between k-Means and the Enhanced k-Means

In this subsection, we will do a performance comparison between the two clustering algorithms, *k*-means, and the enhanced *k*-means, using the dataset (Section 5.1). We will also see how stemming impacts the performance of both clustering algorithms. The comparison will be based on the performance measures purity and the execution time.

**Figure 6:**
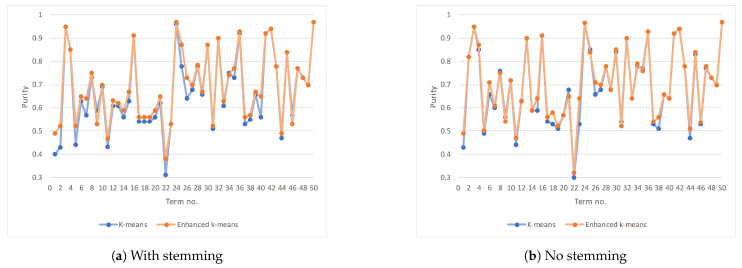
The result’s purity for an individual term on *k*-means and enhanced *k*-means algorithm.

**Figure 7:**
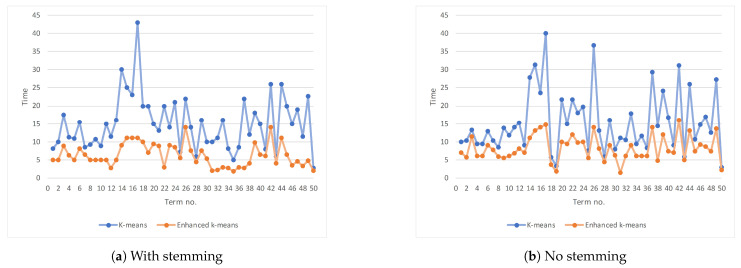
The result’s execution time for an individual term.

Table 8 lists the performance results on selected Arabic terms using the two different clustering algorithms, covering cases when stemming is applied or not. Figure 6a plots the purity for each of the fifty terms where stemming is applied using the two clustering algorithms. Figure 6b is the same but when there is no stemming. Similarly, Figure 7a,b plots the execution time when stemming and no stemming is applied (respectively). Again, we will use the paired *t*-test to confirm if the differences are statistically significant. Our null hypothesis is: there is no difference—on individual performance measures—between both clustering algorithms.

Table 9 summarizes the paired *t*-test for the difference in two performance measures, purity and the execution time. We looked into paired differences of purity between enhanced *k*-means and regular *k*-means for each of the fifty terms under the same condition (i.e., stemming is used or not). Then, we repeated the same method but for the difference in the execution time. In all four cases, we note that $|t|\geq t_{\mathrm{crit}}=2.01$, which means the differences are statistically significant. In other words, the enhanced *k*-means algorithm yields purity, which is significantly better (statistically) than what we achieved using the *k*-means algorithm, regardless of whether stemming is used or not. Moreover, the enhanced *k*-means is faster (statistically significant) than the *k*-means algorithm.

**Figure 8:**
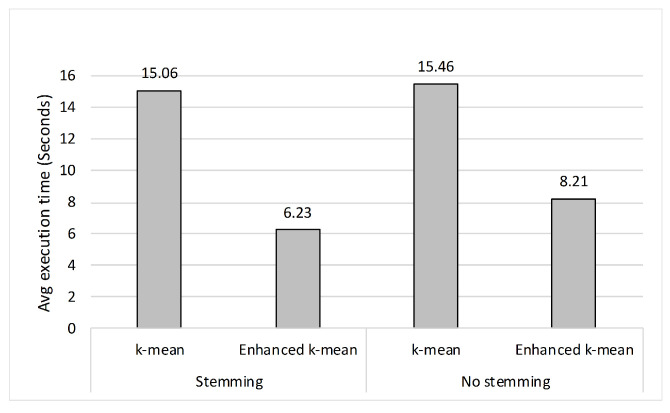
The average clustering execution time.

Figure 8 shows the average execution time for the two clustering algorithms. The enhanced *k*-means algorithm is faster than the regular *k*-means algorithm whether stemming was used or not. When stemming is not used, the average time to cluster using the *k*-means is 15.46 s, which drops to 8.21 s when the enhanced *k*-means is used, and it is 15.06 vs. 6.23 s when stemming is used. This translates to enhanced *k*-means decreased the execution time by 47% when no stemming was used, and by 59% when stemming was used, with a minor improvement in purity.

**Figure 9:**
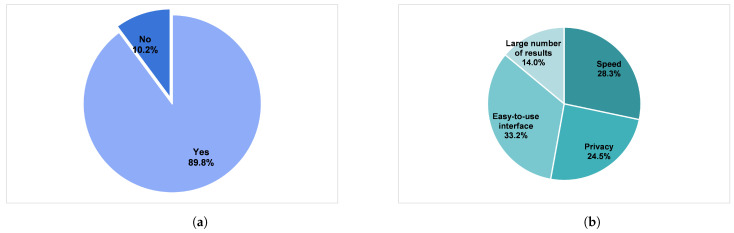
The results of the survey questionnaire: (**a**) choice of search engine that groups the result by topic vs. regular ranked list, and (**b**) criteria for picking search engine. About 90% prefer results clustered by topic. Total of 266 anonymous users surveyed.

We did an anonymous survey on users’ preference for the output of the search engine results page and which criteria they consider the most important when choosing the search engine. For the first question, the choice was between a system that groups the result by topic (i.e., clustered results), and just a regular ranked list (e.g., Google output). Of the 266 respondents, 239 (89.8%) preferred results grouped by topic, with the remaining 10.2% for the normal ranked list. Surely, this goes on to show the frustration most users undergo when doing a regular search. For the second question, there were four choices: (a) speed (how quick the search engine returns the results); (b) easy-to-use interface; (c) privacy; and (d) large number of results. The topmost important factor was an easy-to-use interface (33.2%), followed by speed (28.3%). If we consider the second most important factor, the speed, we can clearly say the users will prefer the enhanced *k*-means algorithm over the regular *k*-means. Figure 9 summarizes the result of the survey.

## 6. Conclusions and Future Work

Many users browse only the top results related to their query, typically those on the first page. Therefore, they might miss relevant documents in traditional IR. This problem can be solved by clustering the search results. Clustering involves grouping similar documents or results into one cluster and labeling it with the most frequent word in the cluster. Clustering the search results helps users quickly find what they are looking for, and it saves time because they can go directly to the right cluster. In this study, we developed an Arabic search result clustering system that extracts Google’s results. The search results text was preprocessed and converted to *tf-idf*, which was later clustered. We then estimated the optimal number of clusters to use by the enhanced *k*-means clustering algorithm. The system clustered the text and generated a cluster label that indicated the content of the cluster. The enhanced *k*-means algorithm results were compared with the results of the regular *k*-means algorithm in both stemmed and non-stemmed texts. The experimental results showed that the enhanced *k*-means algorithm decreased the execution time by 60% for the stemmed dataset and by 47% for the non-stemmed dataset; and in both cases, the purity was slightly improved (statistically significant). Moreover, the results showed that stemming had a slight effect on reducing execution time, and a slight improvement in purity.

For future work, we plan to improve the quality of cluster labels and integrate them into another clustering algorithm. Based on the survey questionnaire, we need to work on improving the user interface of our clustering algorithm.

## Figures and Tables

**Table 1:** Example of an Arabic word that has different affixes attached to a root word, “write”. The full meaning “and I wrote to them”.

ـهم	ت	كاتب	و
*hm*	*t*	*kAtb*	*w*
conjunction	verb	subject pronoun	object pronoun

**Table 2:** List of Arabic terms (and their transliteration) used in our search. Each term has multiple meanings.

(ظهر : *Zhr*)	(عام : *EAm*)	(فطر : *fTr*)	(سبح : *sbH*)	(انف : *Anf*)
(فجر : *fjr*)	(حمام : *HmAm*)	(فك : *fk*)	(شاب : *$Ab*)	(عد : *Ed*)
(كتب : *ktb*)	(هند : *hnd*)	(خال : *xAl*)	(شب : *$b*)	(ناس : *nAs*)
(ذهب : *hb)	(امر : *Amr*)	(كاحل : *kAHl*)	(شرح : *$rH*)	(حاجب : *HAjb*)
(جد : *jd*)	(اذن : A*n)	(لحم : *lHm*)	(سن : *sn*)	(قسم : *qsm*)
(نفسي : *nfsy*)	(بسط : *bsT*)	(لوح : *lwH*)	(سر : *sr*)	(شهد : *$hd*)
(شعر : *$Er*)	(ضل : *Dl*)	(نبع : *nbE*)	(قف : *qf*)	(تولى : *twlY*)
(شال : *$Al*)	(عين : *Eyn*)	(نص : *nS*)	(تحرير : *tHryr*)	(مال : *mAl*)
(ماك : *mAk*)	(فريد : *fryd*)	(نمر : *nmr*)	(تمر : *tmr*)	(طرف : *Trf*)
(شارب : *$Arb*)	(فصل : *fSl*)	(قص : *qS*)	(تسويق : *tswyq*)	(كره : *krh*)

**Table 3:** Summary of evaluating the number of clusters.

	Hard Clustering		Soft Clustering	
	Gap	Gap +	Gap	Gap +
	Statistics	Elbow	Statistics	Elbow
Number of correct answers	19	30	40	47
Accuracy	38%	60%	82%	94%

**Table 4:** Cluster labels for a single term (ذهب : *hb). The score is the term’s weight using the *tf-idf* (term frequency-inverse document frequency) metric.

	Label (Stemmed)		Label (Unstemmed)	
Clus #	Term	Score	Term	Score
0	(ذهب : *hb)	9.991	(الذهب : Al*hb)	10.395
	(اسعار : *AsEAr*)	6.080	(اسعار : *>sEAr*)	5.663
	(عيار : *EyAr*)	3.664	(سعر : *sEr*)	4.223
	(سعر : *sEr*)	3.608	(عيار : *EyAr*)	3.967
	(ثلاثاء : *vlAvA’*)	2.961	(الثلاثاء : *AlvlAvA’*)	3.615
1	(ذهب : *hb)	1.650	(الذهب : Al*hb)	0.899
	(عمل : *Eml*)	1.407	(ذهبت : *hbt)	0.746
	(ولد : *wld*)	1.179	(وقت : *wqt*)	0.668
	(مشاهير : *m$Ahyr*)	1.179	(مسلسل : *mslsl*)	0.650
	(اغنياء : *AgnyA’*)	1.121	(ذهبا : *hbA)	0.631
2	(ذهب : *hb)	0.739	(تعرف : *tErf*)	0.924
	(حديث : *Hdyv*)	0.622	(بياجيه : *byAjyh*)	0.675
	(جرام : *jrAm*)	0.613	(اسود : *Aswd*)	0.592
	(شبكة : *$bkp*)	0.529	(ايقاع : *AyqAE*)	0.577
	(يبلغ : *yblg*)	0.466	(مي : *my*)	0.577

**Table 5:** Performance results on selected Arabic terms using enhanced *k*-means.

Term	Stemming			No Stemming		
	*Purity*	*Entropy*	Time	*Purity*	*Entropy*	Time
كتب	0.95	0.28	8.90	0.95	0.28	11.40
ذهب	0.85	0.47	6.20	0.87	0.38	6.00
شعر	0.64	0.68	6.40	0.61	0.78	7.80
قف	0.56	0.69	2.80	0.54	0.69	14.00
شهد	0.53	0.90	3.45	0.54	0.90	9.18

**Table 6:** The paired *t*-test for the difference in clustering performance with and without stemming on all terms in the dataset. Calculations based on α=0.05. We list SD (standard deviation), SE (standard error), the range for the mean with a 95% confidence interval, *t* (*t* obtained), df (degree of freedom), the 2-tailed *p*-value, *t* critical, and whether the change is significant.

Measure	Paired Differences									
				95% C.I.						
	Mean	SD	SE	Lower	Upper	*t*	df	*p* (2-tailed)	*t*_crit_	Significant
*Purity*	0.0118	0.0626	0.0088	−0.006	0.0296	1.3314	49	0.1892	2.0096	No
*Entropy*	0.0047	0.0671	0.0095	−0.0144	0.0238	0.4935	49	0.6238	2.0096	No
*Time*	1.9798	2.9134	0.4120	1.1518	2.8078	4.8051	49	0.000015	2.0096	Yes

**Table 7:** The distance for selected Arabic terms.

Term	Centroid to Its Datapoints (avg)		Between the Centroids	
	Stemmed	Unstemmed	Stemmed	Unstemmed
عام	71.09	78.16	117.00	153.00
كتب	60.93	66.95	80.50	80.50
بسط	126.56	127.31	211.00	321.50
كاحل	95.29	95.31	146.00	146.00
انف	42.39	61.04	100.50	116.50
سر	63.30	98.05	106.50	159.50

**Table 8:** Performance results on selected Arabic terms using both clustering algorithms.

Terms	Stemming				No Stemming			
	*k*-Means		Enhanced *k*-Means		*k*-Means		Enhanced *k*-Means	
	*Purity*	Time	*Purity*	Time	*Purity*	Time	*Purity*	Time
كتب	0.95	17.46	0.95	8.90	0.95	13.30	0.95	11.40
ذهب	0.85	11.14	0.85	6.20	0.85	9.38	0.87	6.00
شعر	0.57	8.55	0.64	6.40	0.60	10.31	0.61	7.80
قف	0.53	22.00	0.56	2.80	0.53	29.24	0.54	14.00
شهد	0.57	15.00	0.53	3.45	0.53	14.74	0.54	9.18

**Table 9:** The paired *t*-test for the difference in the performance of clustering using two different clustering algorithms with and without stemming on all terms in the dataset. We used α=0.05.

		Paired Diff.					
Meas.	Stem.	Mean	SD	*t*	*p* (2-tail)	*t*_crit_	Signif.
*Purity*	No	0.011	0.024	3.247	0.002	2.01	Yes
	Yes	0.023	0.033	4.673	2.35 × 10^−5^	2.01	Yes
Time	No	−7.256	5.675	−9.042	5.11 × 10^−12^	2.01	Yes
	Yes	−8.829	5.929	−10.53	3.52 × 10^−14^	2.01	Yes

## Data Availability

Data sharing not applicable.
